# Evaluation of Liftover Tools for the Conversion of Genome Reference Consortium Human Build 37 to Build 38 Using ClinVar Variants

**DOI:** 10.3390/genes14101875

**Published:** 2023-09-26

**Authors:** Kyoung-Jin Park, Young Ahn Yoon, Jong-Ho Park

**Affiliations:** 1 Department of Laboratory Medicine & Genetics, Samsung Changwon Hospital, Sungkyunkwan University School of Medicine, Changwon 51353, Republic of Korea; 2 Department of Laboratory Medicine, Soonchunhyang University Cheonan Hospital, Soonchunhyang University College of Medicine, Cheonan 31151, Republic of Korea; motory24@naver.com; 3 Clinical Genomics Center, Samsung Medical Center, Seoul 06351, Republic of Korea; jongho11.park@samsung.com

**Keywords:** ClinVar, Genome Reference Consortium Human Build 37 (GRCh37), GRCh38, liftover, alignment

## Abstract

Although Genome Reference Consortium Human Build 38 (GRCh38) was released with improvement over GRCh37, it has not been widely adopted. Several liftover tools have been developed as a convenient approach for GRCh38 implementation. This study aimed to investigate the accuracy of liftover tools for genome conversion. Two Variant Call Format (VCF) files aligned to GRCh37 and GRCh38 were downloaded from ClinVar (clinvar_20221217.vcf.gz). Liftover tools such as CrossMap, NCBI Remap, and UCSC liftOver were used to convert genome coordinates from GRCh37 to GRCh38. The accuracy of CrossMap, NCBI Remap, and UCSC liftOver were 99.81% (1,567,838/1,570,748), 99.69% (1,565,953/1,570,748), and 99.99% (1,570,550/1,570,748), respectively. Variants that failed conversion via all three liftover tools were all indels/duplications: a pathogenic/likely pathogenic variant (n = 1) and benign/likely benign variants (n = 7). The eight variants that failed conversion were identified in the *ALMS*, *TTN*, *CFTR*, *SLCO*, *LDLR*, *PCNT*, *MID1*, and *GRIA3* genes, and all the variants were not in the VCF files aligned to GRCh37. This study demonstrated that three liftover tools could successfully convert reference genomes from GRCh37 to GRCh38 in more than 99% of ClinVar variants. This study takes the first step to clinically implement GRCh38 using liftover tools. Further clinical studies are warranted to compare the performance of liftover tools and to validate re-alignment approaches in routine clinical settings.

## 1. Introduction

The accuracy and completeness of reference genomes have an important influence on the accuracy of clinical next-generation sequencing (NGS) data analyses. Since the first human reference genome was published in 2001 by the Genome Reference Consortium (GRC), several updated versions of the human reference genome have been released with subsequent incremental improvement (https://www.ncbi.nlm.nih.gov/grc, accessed on 10 August 2023). The most recent build is the Genome Reference Consortium Human Build 38 (GRCh38, released in 2013), which is also referred to as Human Genome 38 (hg38). GRCh38 was released with improvements over GRCh37 (also known as hg19, released in 2009) in terms of accuracy and completeness. Previous studies reported that, compared with GRCh37, GRCh38 updated 8000 nucleotides; filled in numerous gaps; added sequences of centromeres, telomeres, and the mitochondrial genome; and encompassed population-specific genomic contents [1,2].

The different builds of the reference genome not only result in different genome assemblies but also impact genomic analyses and variant classification. Also, the reference allele might not represent the major allele, according to builds, because the reference genome is determined by a very small group of individuals. For instance, the Genome Aggregation Database reports that the allele frequency of the factor V Leiden (FVL) variant [c.1601G > A (p.Arg534Gln) in the *F5* gene] in GRCh37 (Chr1: 169549811) and GRCh38 (Chr1: 169519049) was 98.1% and 1.8%, respectively (https://gnomad.broadinstitute.org/variant/1-169549811-C-T?dataset=gnomad_r3, accessed on 10 August 2023) [3]. The FVL variant may be erroneously defined as the reference allele based on GRCh37. This means that the pathogenic risk alleles based on GRCh38 could be defined as non-pathogenic common alleles based on GRCh37. Furthermore, it has been reported that the use of GRCh37 could result in the false detection of variants in clinically significant genes such as *KCNE1* (Jervell-Lange-Nielsen syndrome 2, long-QT syndrome 5), *NOTCH2* (Alagille syndrome 2; Hajdu-Cheney syndrome), and *SIK1* (developmental and epileptic encephalopathy 30) [2,4]. In addition, sequence differences between GRCh37 and GRCh38 have been reported to be found in disease-related genes such as *NCF1* (Chronic granulomatous disease 1, autosomal recessive), *ADAMTSL2* (Geleophysic dysplasia 1), and *RPS17* (Diamond–Blackfan anemia 4) [2]. Therefore, if GRCh37 continues to be used, there are risks of missing or inaccurately interpreting clinically significant variations. Several studies regarding GRCh38 alignment have reported that the implementation of GRCh38 could produce more accurate and consistent genomic data through the analysis of single nucleotide variations (SNVs), insertions/deletions/duplications (indels/dup), structural variants, and copy number variations, compared with those based on GRCh37 [4,5].

To date, GRCh38 has not been widely adopted and GRCh37 is still extensively used in sequencing data analyses. There are clear benefits and disadvantages of using GRCh38. When switching reference genomes from GRCh37 to GRCh38, the accuracy of NGS data analyses is expected to improve with the improvement of the accuracy and completeness of the reference genome. However, there is a disadvantage in that it is necessary to revalidate the bioinformatic pipeline for the application of GRCh38. This requires additional investment of time, cost, human resources, and computational resources to facilitate the migration. In addition, the raw sequence data for re-alignment can be large, and they are not always accessible. A recent survey demonstrated that clinical laboratories hesitate to migrate to GRCh38; only 7% (2/28) of laboratories migrated to GRCh38 [6]. Furthermore, more than half of the laboratories aligning with GRCh37 (58%, 15/28) responded that they had no plan to change to GRCh38 [6] because their routine bioinformatics pipelines are based on GRCh37, and migration to GRCh38 requires revalidation of the total bioinformatics pipelines [6]. However, if clinical laboratories continuously stick to GRCh37, there would be the risk of missing clinically significant variants as well as the false detection of pathogenic variants [3,4,7,8]. Currently, there is a need for a fast and convenient approach to implement GRCh38.

There are two approaches for reference genome conversion from GRCh37 to GRCh38. One is a time-consuming and computationally expensive re-alignment procedure, and the other is the simple use of liftover tools as an alternative approach to re-alignment [1,9,10,11]. Although re-alignment provides the most accurate results, it may not be practical in clinical laboratories. Several liftover tools such as CrossMap (http://asia.ensembl.org/Homo_sapiens/Tools/AssemblyConverter?db=core, accessed on 10 August 2023), the National Center for Biotechnology Information (NCBI) Remap (https://www.ncbi.nlm.nih.gov/genome/tools/remap, accessed on 10 August 2023), and the University of California Santa Cruz (UCSC) liftOver (https://genome.ucsc.edu/cgi-bin/hgLiftOver, accessed on 10 August 2023), rtracklayer::liftover (https://www.bioconductor.org/help/workflows/liftOver/, accessed on 10 August 2023), flo (https://github.com/wurmlab/flo, accessed on 10 August 2023), and segment_liftover (https://github.com/baudisgroup/segment-liftover, accessed on 10 August 2023) have been developed to convert genome coordinates from one build to another [9,10,11,12,13]. Compared with the alignment approach, liftover has advantages in terms of simplicity and versatility. Liftover tools are easy to use. For example, CrossMap, NCBIRemap, and UCSC liftOver were freely available on the website. In addition, CrossMap and UCSC liftOver only require storage-friendly chain files, which describe pairwise alignment between genome assemblies. Most of the liftover tools also support the most commonly used file types including Browser Extensible Data (BED), General Feature Format (GFF)/Gene Transfer Format (GTF), VCF, Binary Alignment Map (BAM)/Sequence Alignment MAP (SAM), BigWig [9,10,11,12,13]. The liftover tools can be used in any situation where any reference genome conversion is required, including cross-species mapping [9,10,11,12,13].

Different liftover tools can be classified into two categories according to a strategy to preserve the segment: integrity preserved approach (e.g., NCBI Remap) vs. non-integrity preserved approach (e.g., CrossMap, UCSC liftOver, rtracklayer::liftover, flo, and segment_liftover) [9,10,11,12,13]. Currently, the accuracy and limitations of liftover tools are not well known [9,10,11,12,13]. Furthermore, the performance of liftover tools using a large set of clinical variants has not yet been extensively explored [4,13,14,15]. This study aimed to investigate the accuracy of three liftover tools, CrossMap, NCBI Remap, and UCSC liftOver for conversion to GRCh38 from GRCh37, comparing the re-alignment approach.

## 2. Materials and Methods

### 2.1. Statistics of ClinVar Variants

Two VCF files aligned to GRCh37 (No. of clinical variants = 1,573,534) and GRCh38 genome assembly (No. of clinical variants = 1,573,638) were downloaded from ClinVar (clinvar_20221217.vcf.gz). Mitochondrial variants were excluded from the two VCF files [No. of clinical variants = 1,570,644 (from GRCh37) and 1,570,748 (from GRCh38)]. Five variants from the VCF file aligned to GRCh37 were absent in the VCF file aligned to GRCh38, whereas 109 variants from the VCF file aligned to GRCh38 were absent in the VCF file aligned to GRCh37. A total of 1,570,639 variants were in both VCF files aligned to GRCh37 and GRCh38. BED file was generated using position information extracted from the VCF data aligned to GRCh37.

To avoid inconclusive classification, we only selected variants that were provided by multiple submitters with assertion criteria and evidence (2 gold stars), reviewed by an expert panel (3 gold stars), or the variants with practice guideline designation (4 gold stars). The variants (2, 3, or 4 gold stars) were defined as “authentic” ClinVar variants (n = 262,156): SNVs [91.07% (238,735/262,156)] and indels/dup [8.93% (23,421/262,156)]. The classification of the “authentic” ClinVar variants consisted of pathogenic or likely pathogenic (P/LP) variants (n = 42,733), variants of unknown significance (VUS, n = 101,429), benign or likely benign (B/LB) variants (n = 117,826), and others (drug response, other, and “not provided”, n = 105). 

### 2.2. Liftover Tools

The liftover tools such as CrossMap, NCBI Remap, and UCSC liftOver were used to convert genome coordinates from GRCh37 to GRCh38 using the input BED file. To implement CrossMap, a chain format file (GRCh37_to_GRCh38.chain.gz file) was obtained from the following website (https://ftp.ensembl.org/pub/assembly_mapping/homo_sapiens/GRCh37_to_GRCh38.chain.gz, accessed on 10 August 2023). NCBIremap was conducted by remap_api.pl with Assembly-Assembly mode, GCF_000001405.25 (Assembly seqID with GRCh37.p13) source assembly, and GCF_000001405.40 (Assembly seqID with GRCh38.p14) target as-assembly options. UCSC liftover was run on the following website (https://genome.ucsc.edu/cgi-bin/hgLiftOver, accessed on 10 August 2023). The variants on alternative contigs were excluded.

### 2.3. Conversion from GRCh37 to GRCh38

**Figure 1:**
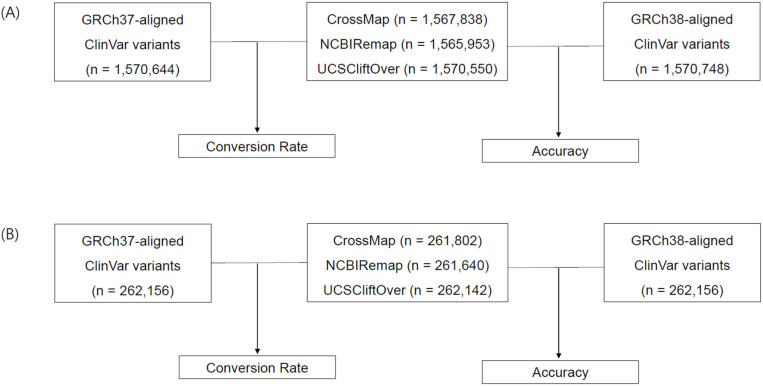
Analysis workflow and variant statistics. The liftover tools CrossMap, NCBI Remap, and UCSC liftOver were used to convert the reference genome from GRCh37 to GRCh38. The conversion rate was calculated based on the comparison between converted variants and GRCh37-aligned variants. The accuracy was assessed based on the comparison between converted variants and GRCh38-aligned variants. Statistics of total ClinVar variants (**A**) and authentic ClinVar variants (**B**).

The conversion rate was defined as the proportion of converted variants from GRCh37 to GRCh38 using liftover tools among aligned variants to GRCh37 (Figure 1, No. of converted variants by CrossMap/No. of GRCh37-aligned variants, No. of converted variants by NCBI Remap/No. of GRCh37-aligned variants, and No. of converted variants by UCSC liftOver/No. of GRCh37-aligned variants). Non-converted variants were defined as the variants that failed conversion, mapped to a different chromosome, or mapped to a different position. Converted variants by using liftover tools were compared to those from ClinVar VCF aligned to GRCh38. The accuracy of the liftover tools was assessed based on ClinVar variants aligned to GRCh38 (Figure 1, No. of converted variants by CrossMap/No. of GRCh38-aligned variants, No. of converted variants by NCBI Remap/No. of GRCh38-aligned variants, and No. of converted variants by UCSC liftOver/No. of GRCh38-aligned variants).

### 2.4. Variant Annotation and Figure Presentation

Variant annotation based on RefSeq transcripts was completed using Ensembl Variant Effect Predictor (VEP release-106). A single identical RefSeq Select was chosen to compare nomenclature between converted variants and aligned variants. Segmental duplications and pseudogenes defined by GENCODE v44 (https://www.gencodegenes.org/human/, accessed on 10 August 2023) were downloaded from the UCSC Table browser (https://genome.ucsc.edu/cgi-bin/hgTables, accessed on 10 August 2023) [16,17]. To investigate whether there are highly homologous sequences in the genomic regions where the variants are located, we annotated the information of segmental duplications and pseudogenes using ANNOVAR (24 October 2019) and bedtools (v2.25.0) [18,19].

Clinical significance of the genes and/or variants was assessed based on the information provided in ClinVar INFO fields and/or Online Mendelian Inheritance in Man (OMIM, https://www.omim.org/downloads, accessed on 10 August 2023).

Figures were presented using Draw Venn Diagram tool (https://bioinformatics.psb.ugent.be/webtools/Venn/, accessed on 10 August 2023) and Microsoft Excel (Microsoft Corporation, Redmond, WA, USA).

## 3. Results

### 3.1. Comparison between GRCh37-Aligned Variants and GRCh38-Aligned Variants

All 5 variants removed from the VCF file aligned to GRCh38 were large indels/dup: NC_000022.10:g.21802791_22555544dup (Variant length of 752,754 bp), NC_000002.11:g.102658576_102847088dup (Variant length of 188,513 bp), NG_009896.1:g.19984_24446dup (Variant length of 5830 bp), NM_005235.2(*ERBB4*):c.83-200864_83-199104del (Variant length of 1761 bp), and NC_000007.14:g.142749126_142753040dup (Variant length of 6503 bp), whereas 109 variants newly included in the VCF file aligned to GRCh38 consisted of a total of 72 indels/dup and 37 SNVs: the variants were in clinically significant genes including *PRAMEF16*, *DHDDS*, *RBM8A*, *CHD1L*, *VPS45*, *NUF2*, *ALMS1*, *ADRA2B*, *TTN*, *IQSEC1*, *RYK*, *DUX4*, *TFAP2B*, *LAMA2*, *FKBP6*, *MUC3A*, *CFTR*, *PRAG1*, *NEFL*, *JRK*, *OPLAH*, *RECQL4*, *PTCH1*, *MUC2*, *PAX6*, *OR8J1*, *MEN1*, *SHANK2*, *ATM*, *C1R*, *LRP6*, *SLCO1B1*, *ALG10*, *BRCA2*, *SPG21*, *SYNM*, *PALB2*, *KCNJ18*, *MAP3K14*, *PPP1R9B*, *PECAM1*, *DSC2*, *ADAMTSL5*, *LDLR*, *CEBPA*, *KLK4*, *ADAM33*, *PRNP*, *LOC102724428*, *PCNT*, *CABIN1*, *MID1*, *WDR45*, *FGF16*, *GRIA3*, *MAMLD1*, *L1CAM*, *MECP2*, *RAB39B*, and *ABO*.

### 3.2. Conversion Rate and Accuracy

**Figure 2:**
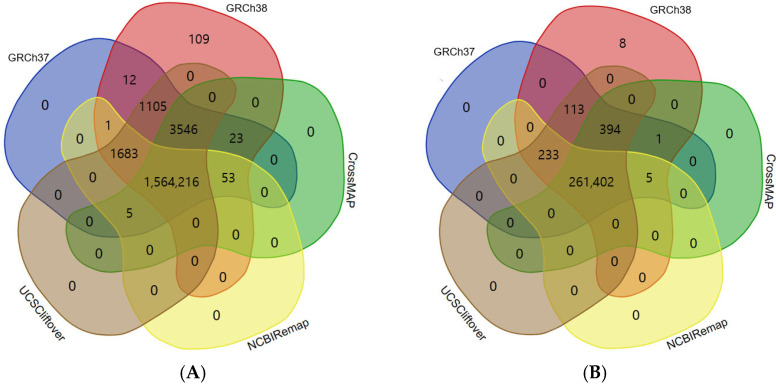
Comparison between aligned data and converted data by liftover tool. Aligned variants were downloaded from the ClinVar database, which consisted of variants aligned to GRCh37 and GRCh38. Converted variants from GRCh37 to GRCh38 were obtained by use of CrossMap, NCBI Remap, and UCSC liftOver. Eight variants that failed conversion by all three liftover tools were not in the VCF file aligned to GRCh37; this means that the variants were in gaps in the reference genome build. Results of total ClinVar variants (**A**) and authentic ClinVar variants (**B**).

**Figure 3:**
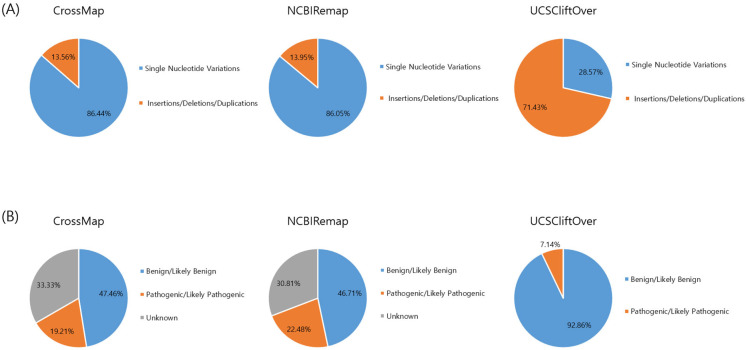
Type and classification of non-converted variants by liftover tool. Type (**A**) and classification (**B**) of the variants.

The conversion rate of CrossMap, NCBI Remap, and UCSC liftOver was 99.82% (1,567,838/1,570,644), 99.70% (1,565,953/1,570,644), and 99.99% (1,570,550/1,570,644), respectively (Figure 1A and Figure 2A). The accuracy of CrossMap, NCBI Remap, and UCSC liftOver was 99.81% (1,567,838/1,570,748), 99.69% (1,565,953/1,570,748), and 99.99% (1,570,550/1,570,748), respectively (Figure 1A and Figure 2A). When analyzing “authentic” clinVar variants, the accuracy of CrossMap, NCBI Remap, and UCSC liftOver was 99.86% (261,802/262,156), 99.80% (261,640/262,156), and 99.99% (262,142/262,156), respectively (Figure 1B and Figure 2B): the accuracy and conversion rates of “authentic” variants were the same, because “authentic” variants were shared in both VCF files aligned to GRCh37 and GRCh38. The proportion of indels/dup among non-converted variants by CrossMap, NCBI Remap, and UCSC liftOver were 13.56% (48/354), 13.95% (72/516), and 71.43% (10/14), respectively (Figure 3, Supplementary Tables S1–S3). The variants which failed conversion by all three liftover tools were indels/dup: NM_001378454.1(*ALMS1*):c.1571CTC[1] (p.Pro525del), NM_001267550.2(*TTN*):c.100766-10dup, NM_000492.3(*CFTR*):c.1210-12T[9], NM_006446.4(*SLCO1B1*):c.359+10[16], NM_000527.5(*LDLR*):c.314-446_1187-386dup, NM_006031.6(*PCNT*):c.8751+23dup, NM_000381.4(*MID1*):c.661-7dup, and NM_000828.4(*GRIA3*):c.-2G=. These non-converted variants were not in the VCF file aligned to GRCh37 (Figure 2B).

The frequency of P/LP variants among variants that failed conversion by any liftover tools was 23.47% (177/754): 19.21% (68/354) for CrossMap, 22.48% (116/516) for NCBI Remap, and 7.14% (1/14) for UCSC liftOver, respectively (Figure 3, Supplementary Tables S1–S3). The non-converted P/LP variants by CrossMap (n = 68) were identified in *GPR179* (n = 5), *RBP3* (n = 3), *ZNHIT3* (n = 1), *HNF1B* (n = 57), *LDLR* (n = 1), and *PCGF2* (n = 1) genes (Supplementary Table S1). The non-converted P/LP variants by NCBI Remap (n = 116) were identified in the *DGAT1* (n = 2), *GPR179* (n = 5), *LDLR* (n = 1), *MBOAT7* (n = 1), *PQBP1* (n = 7), *PRPF31* (n = 29), *PUF60* (n = 7), *SLC35A2* (n = 3), *SLC39A4* (n = 2), *SLC52A2* (n = 5), *SURF1* (n = 26), *TFE3* (n = 2), *WDR45* (n = 25), *ZNHIT3* (n = 1) genes (Supplementary Table S2). The non-converted P/LP variant by UCSC liftOver (n = 1) was in the *LDLR* (n = 1) gene (Supplementary Table S3). The P/LP variant which failed conversion by all three liftover tools was NM_000527.5(*LDLR*):c.314-446_1187-386dup (Supplementary Tables S1–S3).

## 4. Discussion

We have evaluated three liftover tools for genome conversion using clinical variants from ClinVar. The ClinVar is one of the most commonly used clinical databases for variant curation and interpretation. Currently, studies regarding genome conversion for clinical variants using liftover tools have not been rigorously investigated. Pan et al., have reported the conversion rate (average 99%) of SNV from NA12878 from GRCh37 to GRCh38 using LiftoverVcf from the Picard package and CrossMap [14]. Ormond et al. have investigated the conversion failure rates of 0.14% from GRCh37 to GRCh38 [15]. These two studies have been performed using reference materials such as NA12878, and they do not represent clinical variants [14,15]. In addition, previous studies focused on the conversion of SNVs and did not consider indels/dup [14,15]. To date, there has been only one study regarding the genome conversion of a limited set of clinical variants (n = 158) using UCSC liftOver [4]. We investigated the conversion rate and accuracy of liftover tools using a large set of clinical variants from ClinVar (GRCh37-aligned ClinVar variants and GRCh38-aligned ClinVar variants) including indels/dup as well as SNVs.

There were significant discrepancies between GRCh37-aligned ClinVar variants and GRCh38-aligned ClinVar variants. We found that indels/dup were added more in the GRCh38-aligned VCF file [(indels/dup (n = 72) vs. SNVs (n = 37))]. That means that using GRCh38 can produce more indels/dup compared to GRCh37. A number of variants newly included in the GRCh38-aligned VCF file were in clinically significant genes, including *RBM8A* (Thrombocytopenia-absent radius syndrome), *CHD1L* (HCCs and other solid tumors), *NUF2* (4p partial monosomy syndrome), *ALMS1* (Alstrom syndrome), *TTN* (Tibial muscular dystrophy, Myopathy, myofibrillar, 9, with early respiratory failure, Autosomal recessive limb-girdle muscular dystrophy type 2J, Early onset myopathy with fatal cardiomyopathy), *TFAP2B* (Chronic intestinal pseudo-obstruction), *LAMA2* (Merosin deficient congenital muscular dystrophy), *FKBP6* (Male infertility, Spermatogenic failure 77), *MUC3A* (Lung cancer), *CFTR* (Cystic fibrosis, CFTR-related disorders, Obstructive azoospermia, Hereditary pancreatitis, Congenital bilateral aplasia of vas deferens from CFTR mutation, Bronchiectasis with or without elevated sweat chloride 1, modifier of), *NEFL* (Charcot-Marie-Tooth disease type 2E), *OPLAH* (5-Oxoprolinase deficiency), *RECQL4* (Baller-Gerold syndrome), *PTCH1* (Gorlin syndrome), *MUC2* (Small cell lung carcinoma), *PAX6* (Aniridia 1), *OR8J1* (Premature ovarian failure), *MEN1* (Hereditary cancer-predisposing syndrome), *SHANK2* (Autism spectrum disorder), *ATM* (Ataxia-telangiectasia, Lymphoma), *C1R* (Ehlers-Danlos syndrome, periodontal type 2), *LRP6* (Ductal breast carcinoma), *SLCO1B1* (Hyperbilirubinemia, Rotor type, digenic), *ALG10* (Delayed puberty), *BRCA2* (Hereditary breast ovarian cancer syndrome), *PALB2* (Hereditary cancer-predisposing syndrome), *KCNJ18* (Thyrotoxic periodic paralysis, susceptibility to, 2), *MAP3K14* (NIK deficiency), *PECAM1* (Three Vessel Coronary Disease), *DSC2* (Arrhythmogenic right ventricular dysplasia 11), *ADAMTSL5* (Ductal breast carcinoma), *LDLR* (Hypercholesterolemia, familial, 1), *CEBPA* (Acute myeloid leukemia), *KLK4* (Amelogenesis imperfecta), *ADAM33* (Ductal breast carcinoma), *PRNP* (Inherited Creutzfeldt-Jakob disease, Gerstmann–Straussler–Scheinker syndrome, Huntington disease-like 1), *LOC102724428* (Childhood epilepsy with centrotemporal spikes), *PCNT* (Microcephalic osteodysplastic primordial dwarfism type II), *MID1* (Opitz GBBB syndrome), *WDR45* (Neurodegeneration with brain iron accumulation 5), *FGF16* (Metacarpal 4-5 fusion), *GRIA3* (Syndromic X-linked intellectual disability 94), *MAMLD1* (Disorder of sexual differentiation), *L1CAM* (X-linked hydrocephalus syndrome), *MECP2* (Encephalopathy, neonatal severe, Rett syndrome), *RAB39B* (Early onset parkinsonism-intellectual disability syndrome), and *ABO* (ABO blood group system). When limited to “authentic” ClinVar variants, we showed that only eight variants, which were newly added in the GRCh38-aligned VCF file and were not in GRCh37-aligned VCF, failed conversion by all three liftover tools. The presence of these variants may attribute false negative results when using liftover tools. Because we implemented the liftover tools using GRCh37-aligned ClinVar variants, it is reasonable that these variants were not detected as converted variants by liftover tools. To minimize false negatives, differences between reference genome builds should be considered ahead of the performance of liftover tools, because these variants are likely to be in gaps between the reference genome builds. Compared to GRCh37, GRCh38 updated 8000 nucleotides, corrected several misassembled regions, and filled in numerous gaps, indicating an ability to detect clinically significant variants with higher sensitivity [1,2].

To the best of our knowledge, there are no studies regarding the comparison of three liftover tools using clinical variants. One recent study has reported a high degree of correlation between liftover tools including CrossMap, NCBI Remap, and UCSC liftOver using epigenetic data such as DNA methylation and Chromatin ImmunoPrecipitation Sequencing data [13]. In this study, we showed the similar accuracy of liftover tools using genetic data; three liftover tools could successfully convert reference genomes from GRCh37 to GRCh38 in more than 99% of ClinVar variants [CrossMap (99.82%), NCBI Remap (99.70%), and UCSC liftOver (99.99%)].

Here, we provided a list of non-converted P/LP variants identified in clinically significant genes such as *DGAT1* (Congenital diarrhea 7 with exudative enteropathy), *GPR179* (Congenital stationary night blindness), *HNF1B* (Maturity onset diabetes mellitus in young, Renal cysts and diabetes syndrome), *LDLR* (familial hypercholesterolemia), *MBOAT7* (Intellectual disability, autosomal recessive), *PCGF2* (Abnormality of the outer ear, Intellectual disability, Global developmental delay, Turnpenny-fry syndrome), *PQBP1* (Renpenning syndrome, Microcephaly, Intellectual disability, Delayed speech and language development, Hyperactivity), *PRPF31* (Retinal dystrophy, Retinitis pigmentosa), *PUF60* (8q24.3 microdeletion syndrome, CHARGE association), *RBP3* (Retinal dystrophy, Retinitis pigmentosa), *SLC35A2* (non-lesional focal epilepsy, SLC35A2-congenital disorder of glycosylation), *SLC39A4* (Hereditary acrodermatitis enteropathica), *SLC52A2* (Brown-Vialetto-van Laere syndrome 2), *SURF1* (Cerebellar ataxia, Dysarthria, Muscle weakness, Abnormal pyramidal sign, Cytochrome-c oxidase deficiency disease, Charcot-Marie-Tooth disease type 4K, Leigh syndrome), *TFE3* (Neurodevelopmental abnormality), *WDR45* (Delayed speech and language development, development, Neurodegeneration with brain iron accumulation 5, X-linked cerebral-cerebellar-coloboma syndrome), *ZNHIT3* (PEHO syndrome) (Supplementary Tables S1–S3). There is a higher risk of missing clinically significant variants of these genes in clinical laboratories that remain with the use of GRCh37 as a reference genome.

However, most of the P/LP variants, except one, were converted successfully by the combined use of multiple liftover tools. One P/LP variant of NM_000527.5(*LDLR*):c.314-446_1187-386dup, which failed conversion by all three liftover tools, was an 8.1kb duplication variant. Considering the size of the variant, the direct re-alignment approach is more appropriate than using liftover tools for this variant; the larger the genomic segments and intervals of the variant, the lower the accuracy result of the liftover tools for genome conversion of the variant.

Here, we demonstrated that most of the non-converted variants by a single liftover tool were successfully converted by the other tools. For example, the variant of NM_002900.3(*RBP3*):c.1682_1686dup (p.Thr563fs) was not converted by CrossMap, whereas it was converted successfully by NCBI Remap and UCSC liftOver. Another example of NM_006331.8(*EMG1*):c.126dup (p.Leu43fs) was not converted by UCSC liftOver, whereas it was converted successfully by NCBI Remap and CrossMap. This suggested that the combined use of multiple liftover tools might increase the conversion rate and accuracy of the liftover tools.

There are pros and cons of using multiple liftover tools simultaneously. As shown in this study, the simultaneous use of multiple liftover tools could result in a more successful genome conversion and they could be used complementarily with each other. Fortunately, UCSC liftOver, NCBI Remap, and CrossMap are freely available on the public web. Therefore, these tools can be easily accessed, even in laboratories without bioinformatics resources. However, most liftover tools also require chain files or alignment formats and other liftover tools are relatively time consuming and computationally intensive, although they are a relatively convenient and more cost-effective approach than re-alignment [12,13]. Currently, the accuracy and limitations of these liftover tools have not been extensively studied. Therefore, further clinical studies should be performed to investigate the clinical utility as well as analytical utility of several liftover tools in routine clinical settings prior to the simultaneous use of liftover tools.

We investigated the type of non-converted variants. We found that the proportion of indels/dup among the total studied variants was 8.93% (23,421/262,156), while the proportion of indels/dup among non-converted variants by CrossMap, NCBI Remap, and UCSC liftOver was 13.56% (48/354), 13.95% (72/516), and 71.43% (10/14), respectively. Indels/dup are more complicated in variant size and sequence context and are likely to be more error-prone than SNVs. In addition, indels/dup frequently occur in repetitive sequences, and this can make analyses difficult. Therefore, when using the liftover tools, especially UCSC liftover, it may be helpful to consider the type of variants.

Next, we performed variant annotation and investigated whether there are highly homologous sequences such as pseudogenes and segmental duplications in the genomic regions where the variants are located. Considering that liftover tools “lift” the genome position in one reference genome build ‘over’ to another build, highly homologous sequences might also result in genome conversion failure. However, in this study, we found no pseudogene-related factors in any non-converted variants among authentic ClinVar variants.

Previous studies have investigated the problems associated with genome conversion using liftover tools [13,14,15]. Pan et al., have shown that discordant SNVs had lower read depth and a higher prevalence of GC contents [14]. Ormond et al., have reported that conversion-unstable positions were associated with gaps in the builds, contig differences between builds, and segmental duplications [15]. Therefore, it is important to pre-exclude the problematic regions such as gapped regions before the implementation of liftover tools. Here, we pre-excluded the variants on alternative contigs to exclude conversion failure due to contig differences between builds. In the present study, we did not investigate comprehensively the genomic regions associated with conversion failure by liftover tools because we analyzed only VCF files downloaded from ClinVar. Neither FASTQ or BAM files were available in this study. We demonstrated that gaps in the reference builds or variant types such as indels/dup were associated with conversion failure. Further clinical studies to investigate the genomic characteristics associated with conversion failure would be recommended prior to the clinical use of liftover tools.

Previous studies reported that converted variants using liftover tools are not always concordant with the variants obtained from the aligned data [13,14,15]. These variants might be false positive results of liftover tools because these variants were not in the VCF file obtained from alignments to GRCh38, which is considered as ground truth. In this study, five variants that were included in the GRCh37-aligned VCF file and removed from the GRCh38-aligned VCF file [for example, NM_005235.2(*ERBB4*):c.83-200864_83-199104del] were also successfully converted by all three liftover tools. Despite successful conversion, these variants should not be reported if GRCh38 was chosen as a reference genome. Here, there was no discordant variants among the authentic ClinVar variants. Previous studies reported that discordant variants were noted to be in regions with segmental duplications and repetitive sequences [13,14,15]. Furthermore, it has been reported that there were changes in the chromosome and genomic position of converted variants by use of liftover tools [10,14,15]. Inconsistent chromosome numbers between reference genomes by different liftover tools can negatively impact downstream analyses in terms of nomenclature and classification of variants. Another explanation for discordancy is that the position in the GRCh37 is not in the GRCh38 or vice versa [13,14,15]. This is because all positions are not completely comparable between the reference genomes.

## 5. Conclusions

In conclusion, three liftover tools could successfully convert reference genomes from GRCh37 to GRCh38 in more than 99% of ClinVar variants. We showed that gaps in the reference builds and variant types such as indels/dup were associated with conversion failure using liftover tools. In addition, we provided the list of non-converted P/LP variants from GRCh37 to GRCh38 by liftover tools. This list can be used to pre-exclude the variants and/or genes prior to the implementation of liftover tools. The liftover tools might be one of the practical alternatives for genome conversion in case re-alignment approaches were not possible, even if they do not guarantee a completely accurate conversion. The use of multiple liftover tools and pre-excluding of known variants in conversion failure regions before the implementation of liftover tools could result in more successful genome conversion. To our knowledge, this is the first study regarding accuracy of three liftover tools using the largest set of clinical variants. This study takes the first step for the clinical implementation of GRCh38. Further clinical studies are warranted to validate the performance of liftover tools, to characterize the non-converted variants in routine clinical settings, and, eventually, to improve the accuracy of liftover tools.

## Data Availability

The ClinVar variants analyzed in this study are available at https://ftp.ncbi.nlm.nih.gov/pub/clinvar/ (accessed on 22 December 2022).

## Supplementary Materials

The following supporting information can be downloaded at: https://www.mdpi.com/article/10.3390/genes14101875/s1. Table S1. Pathogenic and/or likely pathogenic variants among variants which failed conversion by CrossMap. Table S2. Pathogenic and/or likely pathogenic variants among variants which failed conversion by NCBI Remap. Table S3. Pathogenic and/or likely pathogenic variants among variants which failed conversion by UCSC liftOver.

## References

[1] Guo Y., Dai Y., Yu H., Zhao S., Samuels D.C., Shyr Y. (2017). Improvements and impacts of GRCh38 human reference on high throughput sequencing data analysis. Genomics.

[2] Schneider V.A., Graves-Lindsay T., Howe K., Bouk N., Chen H.C., Kitts P.A., Murphy T.D., Pruitt K.D., Thibaud-Nissen F., Albracht D. (2017). Evaluation of GRCh38 and de novo haploid genome assemblies demonstrates the enduring quality of the reference assembly. Genome Res..

[3] Karczewski K.J., Francioli L.C., Tiao G., Cummings B.B., Alfoldi J., Wang Q., Collins R.L., Laricchia K.M., Ganna A., Birnbaum D.P. (2020). The mutational constraint spectrum quantified from variation in 141,456 humans. Nature.

[4] Lansdon L.A., Cadieux-Dion M., Herriges J.C., Johnston J., Yoo B., Alaimo J.T., Thiffault I., Miller N., Cohen A.S.A., Repnikova E.A. (2022). Clinical Validation of Genome Reference Consortium Human Build 38 in a Laboratory Utilizing Next-Generation Sequencing Technologies. Clin. Chem..

[5] Li H., Dawood M., Khayat M.M., Farek J.R., Jhangiani S.N., Khan Z.M., Mitani T., Coban-Akdemir Z., Lupski J.R., Venner E. (2021). Exome variant discrepancies due to reference-genome differences. Am. J. Hum. Genet..

[6] Lansdon L.A., Cadieux-Dion M., Yoo B., Miller N., Cohen A.S.A., Zellmer L., Zhang L., Farrow E.G., Thiffault I., Repnikova E.A. (2021). Factors Affecting Migration to GRCh38 in Laboratories Performing Clinical Next-Generation Sequencing. J. Mol. Diagn..

[7] Press R.D., Bauer K.A., Kujovich J.L., Heit J.A. (2002). Clinical utility of factor V leiden (R506Q) testing for the diagnosis and management of thromboembolic disorders. Arch. Pathol. Lab. Med..

[8] Bhatt S., Taylor A.K., Lozano R., Grody W.W., Griffin J.H., Practice A.P., Guidelines C. (2021). Addendum: American College of Medical Genetics consensus statement on factor V Leiden mutation testing. Genet. Med..

[9] Coordinators N.R. (2018). Database resources of the National Center for Biotechnology Information. Nucleic Acids Res..

[10] Kuhn R.M., Haussler D., Kent W.J. (2013). The UCSC genome browser and associated tools. Brief. Bioinform..

[11] Zhao H., Sun Z., Wang J., Huang H., Kocher J.P., Wang L. (2014). CrossMap: A versatile tool for coordinate conversion between genome assemblies. Bioinformatics.

[12] Pracana R., Priyam A., Levantis I., Nichols R.A., Wurm Y. (2017). The fire ant social chromosome supergene variant Sb shows low diversity but high divergence from SB. Mol. Ecol..

[13] Luu P.L., Ong P.T., Dinh T.P., Clark S.J. (2020). Benchmark study comparing liftover tools for genome conversion of epigenome sequencing data. NAR Genom. Bioinform..

[14] Pan B., Kusko R., Xiao W., Zheng Y., Liu Z., Xiao C., Sakkiah S., Guo W., Gong P., Zhang C. (2019). Similarities and differences between variants called with human reference genome HG19 or HG38. BMC Bioinform..

[15] Ormond C., Ryan N.M., Corvin A., Heron E.A. (2021). Converting single nucleotide variants between genome builds: From cautionary tale to solution. Brief. Bioinform..

[16] Frankish A., Diekhans M., Jungreis I., Lagarde J., Loveland J.E., Mudge J.M., Sisu C., Wright J.C., Armstrong J., Barnes I. (2021). Gencode 2021. Nucleic Acids Res..

[17] Kent W.J., Sugnet C.W., Furey T.S., Roskin K.M., Pringle T.H., Zahler A.M., Haussler D. (2002). The human genome browser at UCSC. Genome Res..

[18] Wang K., Li M., Hakonarson H. (2010). ANNOVAR: Functional annotation of genetic variants from high-throughput sequencing data. Nucleic Acids Res..

[19] Quinlan A.R., Hall I.M. (2010). BEDTools: A flexible suite of utilities for comparing genomic features. Bioinformatics.

